# Diagnosis and Management of Trochleodynia, Trochleitis, and Trochlear Headache

**DOI:** 10.3389/fneur.2019.00361

**Published:** 2019-04-12

**Authors:** Tu M. Tran, Collin M. McClelland, Michael S. Lee

**Affiliations:** ^1^Department of Ophthalmology and Visual Neurosciences, University of Minnesota, Minneapolis, MN, United States; ^2^Department of Neurology, University of Minnesota, Minneapolis, MN, United States; ^3^Department of Neurosurgery, University of Minnesota, Minneapolis, MN, United States

**Keywords:** trochleodynia, trochleitis, trochlear, headache, Brown syndrome, corticosteroid, treatment, diagnosis

## Abstract

Migraine and tension-type headaches (TTHs) comprise a significant burden of neurological disease globally. Trochleodynia, also known as primary trochlear headache or trochleitis, may go unrecognized and contribute to worsening of these headache disorders. It may also present in isolation. We review the English literature on this under-recognized condition and describe what is known about the theorized pathophysiology, clinical presentation, and differential diagnosis. We also present a management algorithm for patients presenting with trochleodynia.

## Introduction

Migraine headache (MH) occurs in 15.3–16.0% of adults in the United States and Europe, and 73.2% individuals with chronic migraines report moderate to severe disability (1–3). MH and tension-type headache (TTH) account for 14.0% of the total global burden of neurological diseases, based on disability-adjusted life years (DALYs) (4). Trochlear pain may represent a source of exacerbation and treatment failure in MHs and TTHs or it may occur in isolation. Diagnosis and early treatment could lead to better outcomes and reduced level of disability in patients suffering from poorly controlled MH or TTH. Meanwhile, trochlear pain treatment is commonly overlooked, since most patients often have coexisting headache disorders (5, 6).

**Figure 1:**
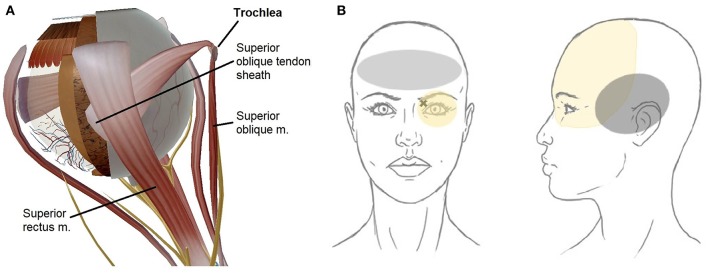
Schematic of trochlear region anatomy (created using Human Anatomy Atlas 8, Visible Body, Boston, MA, USA). **(A)** Superior oblique tendon sheath covering the tendon hidden from view. **(B)** Trochlear region depicting periocular pain distribution (in yellow). The X marks the superomedial orbit with highest focal tenderness, typical front-parietal topography. Left panel (gray area): Bilateral frontal predominant pain suggests comorbid tension-type headache, which can also be triggered by trochleodynia. Right panel (gray area): Temporal location maybe indicative of comorbid migraine headache which can be triggered by trochleodynia; migraine headaches typically involve unilateral ocular and frontal regions as well.

Here, we categorize trochleodynia as a spectrum of disorders characterized by pain arising from the trochlear region and one or more of the following structures: the cartilaginous trochlea, the superior oblique (SO) muscle, the SO tendon and fibrovascular sheath, and the surrounding nerves that provide nociceptive input, mainly the supraorbital and supratrochlear nerves (Figure 1) (5). The literature on this entity is scarce, but recently, it has become recognized as a distinct disorder by the International Headache Society (7) and case series have arisen from Italy (8), Spain (9), Thailand (10), and United States (6). Herein, we use the term, trochleodynia, as a clinical diagnosis that encompasses what has been previously described as trochleitis (11) and primary trochlear headache (PRTH) (12). Although trochleitis is thought to have an inflammatory etiology while primary trochlear headache does not, we agree with the International Classification of Headache Disorders (ICHD) that both entities can be lumped together under the overarching diagnosis of trochleodynia, since the presentation and treatment is similar for both entities. Trochleodynia has also been associated with Brown syndrome (Brown syndrome associated with trochleodynia—BSAT), which has added sequela of fibrosis resulting in ophthalmoplegia (8). Trochleodynia's prevalence was estimated at 12 per 100,000 in one retrospective cohort from 2003 to 2010, though this may be an underestimation due to limited awareness of the diagnosis in the past (9).

As recognition of trochleodynia grows, there is an imperative to better manage these disorders. Using published case series and case reports, we review the diagnostic criteria, management considerations, and propose an algorithm for evaluation and management.

### Pathophysiology

There are three probable etiologies of the pain experienced in trochleodynia: (1) neuropathic, (2) neuromuscular, and (3) inflammatory. The neuropathic pain hypothesis was first proposed by Yanguela et al. in a case series of 18 patients (5). A cycle of repeated trauma to the supraorbital and supratrochlear nerve running proximal to the trochlea leads to nociception perceived in the periorbital or frontal hemicranial distribution. This hypothesis is supported by a case of a 27 year-old male with SO myokymia (SOM) (13) who subsequently developed trochleodynia. The authors believed that repeated SO contraction could have led to the cycle of trauma to the supraorbital and supratrochlear nerves.

The neuromuscular etiology is of particular relevance to MH and TTH patients since it is established that myofascial trigger points (MTrP) are more prevalent in affected individuals compared to controls and likely play a significant role in the pathophysiology (14). A MTrP is a hyperirritable location in skeletal muscle associated with a taut myofascial bundle. Provocation of a MTrP may exacerbate sensory nerve injury or induce nerve entrapment. MTrp in the SO muscle (15, 16) leads to increased frequency of nociceptive input from supraorbital or supratrochlear nerves toward the spinal trigeminal nucleus caudalis (17).

Finally, inflammation is the most well-characterized etiology of pain. In 1984, Tychsen first demonstrated histopathologic features of perivascular lymphocytic infiltration of connective and adipose tissue adjacent to trochlear cartilage with invasion of the SO myofibrils in a patient with trochleodynia who underwent biopsy (11). The inflammatory process is most often idiopathic and manifests unilaterally. Bilateral inflammation is almost always secondary to a systemic inflammatory condition, such as incomplete Behçet's syndrome (6), granulomatosis with polyangiitis (GPA) (6), systemic lupus erythematosus (SLE) (18), and adult-onset Still's disease (AOSD) (19). For unclear reasons, bilateral inflammation often presents sequentially with a variable delay between ipsilateral presentation and eventual bilateral involvement (6, 10). Bilateral idiopathic inflammation is rare and has only been reported once to our knowledge (20). Other etiologies of trochleodynia have been considered iatrogenic but still inflammatory in nature, for example, one case reported after optic nerve sheath meningioma resection and another case following orbital decompression for Grave's ophthalmopathy (6). Interestingly, one patient developed trochleodynia 1 month after Roux-en-Y gastric bypass (6), although it remains unclear whether the two are related.

Neuropathic and neuromuscular pain may be the underlying etiology for PRTH and inflammation may be primary pathology in trochleitis, but there is likely overlap in the pathophysiology of these entities. More research may eventually yield new paradigms in diagnosis and management, but at this moment, they do not differ clinically enough to consider PRTH and trochleitis as two separate clinical diagnoses.

## Diagnostic Considerations

### Clinical Presentation

Clinical signs include tenderness in the trochlear region and exacerbation of pain with SO muscle contraction or stretching from eye movement or near-work (e.g., reading, computer, sewing, etc.). Despite a proposed inflammatory component, patients do not typically exhibit eyelid edema or erythema. Patients often point to the affected trochlear area when asked about location The pain is often continuous with episodic exacerbations. The pain is characteristically severe, commonly endorsed at ranges of 7–10 out of 10 on the visual analog scale in 20.8% (95%CI 11.4–35.0) of cases (5, 6, 12). The pain increases with trochlear palpation (3–4 points higher on the visual analog scale); SO stretching from elevation in abduction or SO contraction by depression in adduction may increase pain perception outside the trochlear region (4–5 points higher) (15, 16). Extra-trochlear pain is usually described as retro-orbital or supra-orbital.

Transient or constant diplopia in primary gaze rarely occurs in 5% or less of cases (10). Most patients note ipsilateral pain which radiated bilaterally in about half (51.9%, 95%CI 42.3–61.4) of 104 published cases (5, 6, 10–12).

**Figure 2:**
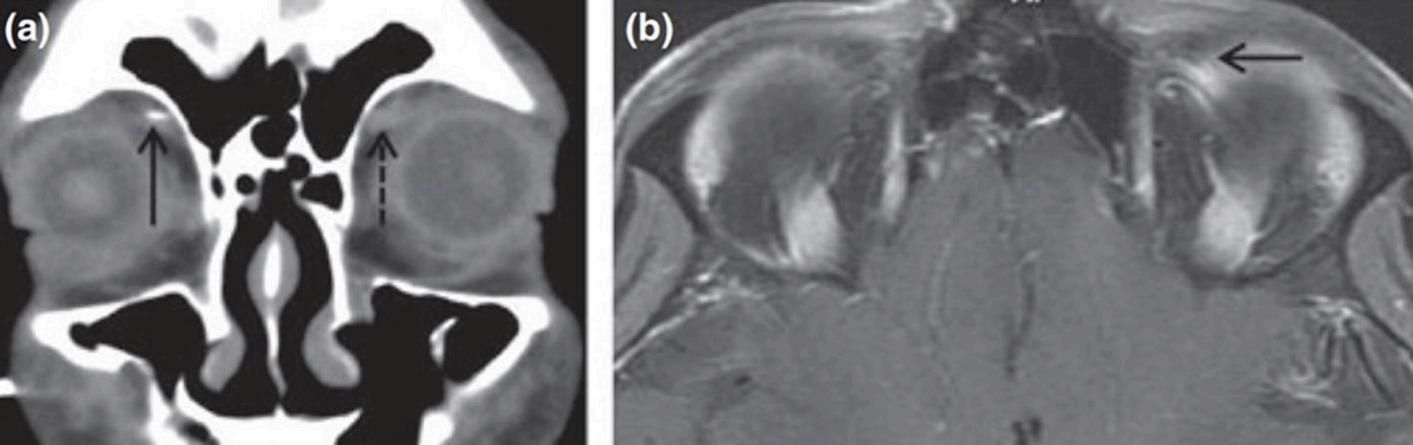
Computed tomography and magnetic resonance imaging findings of trochlear inflammation [adapted from Smith et al. (6) with permission granted by John Wiley and Sons]. **(a)** Coronal CT showing soft tissue enhancement of left trochlea (broken arrow) from acute inflammation. **(b)** MRI of orbits with gadolinium enhancement showing uptake at the left trochlea (solid arrow).

Trochleodynia is predominately a clinical diagnosis. If necessary, imaging can be used to confirm trochlear inflammation, while ruling out more serious diagnoses, such as other orbital or cavernous sinus disorders. When the inflammation is marked and diffuse, computed tomography (CT) and magnetic resonance imaging (MRI) scans may show characteristic findings (Figure 2). Given the small size of the trochlea and the inherent resolution limitations of CT and MRI, lack of radiographic evidence of inflammation does not exclude a diagnosis of trochleodynia. Among 115 combined patients, only 20% had CT or MRI abnormalities (5, 6, 8–10, 12, 18–22), suggesting that while characteristic imaging findings of inflammation may secure an otherwise unclear diagnosis of trochleodynia, it is not required.

Secondary trochleodynia related to underlying systemic conditions varies in presentation. In one case of GPA (6), trochleodynia was the presenting symptom that led to the workup and eventual diagnosis. In another case, a 26 year-old woman with SLE presented with trochleodynia 27 months prior to onset of alopecia, arthralgias, livedo reticularis, Raynaud's, and nailbed abnormalities (18). In a 23 year-old male with AOSD, the symptom onset occurred at the same time as systemic symptoms; trochleodynia symptoms remitted once the patient was diagnosed correctly with AOSD and treated with anakinra, an IL-1 receptor antagonist. Evaluation for connective tissue inflammatory diseases should be considered if a patient presents bilaterally, especially if there are any constitutional symptoms or signs. Some propose unilateral cases should be worked up as well considering there is often a temporal gap between unilateral presentation and contralateral involvement in bilateral cases. If clinical suspicion is high enough, workup for underlying inflammatory diseases should follow and may include tests listed in Table 1.

**Table 1:** Basic laboratory workup in evaluation of trochleodynia.

**Test**	**Findings of interest**
Complete blood count	Systemic inflammatory disease screen
Hemostasis	Systemic inflammatory disease screen
Urinalysis	Systemic inflammatory disease screen
Erythrocyte sedimentation rate/C-reactive protein	Systemic inflammatory disease screen
Thyroid panel, thyroid stimulating immunoglobulin	Thyroid eye disease
Chest X-ray, serum ACE	Sarcoidosis
Electrocardiogram	Undifferentiated connective tissue disease screen
Anti-nuclear antibodies	Undifferentiated connective tissue disease screen
Rheumatoid factor	Rheumatoid arthritis, Systemic lupus erythematosus, Undifferentiated connective tissue disease screen
Anti-dsDNA	Systemic lupus erythematosus
Anti-SSA (Ro), Anti-SSB (La)	Sjögren's syndrome
Anti-ANCA antibodies	Granulomatosis with polyangiitis, Microscopic polyangiitis, Eosinophilic granulomatosis with polyangiitis
HIV, RPR/VDRL, and FTA-ABS, Lyme, QuantiFERON-Tb, bacterial and fungal cultures MRI brain and orbit with contrast	Infectious workup if suspecting cavernous sinus syndrome

### Brown Syndrome Associated With Trochleodynia (BSAT)

**Figure 3:**
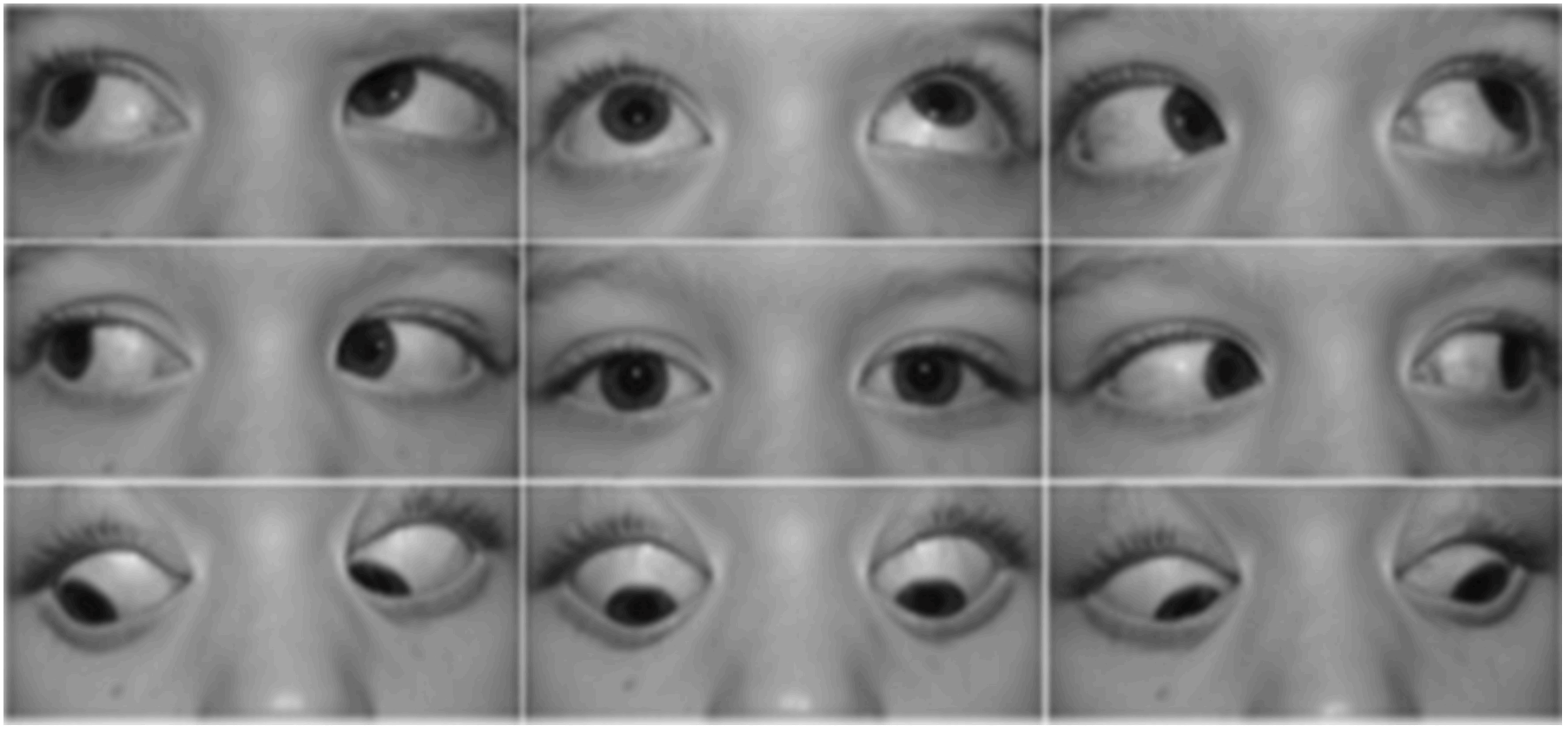
Representative supraduction in adduction deficit in the right eye due to acquired Brown syndrome [reproduced from Giannaccare et al. (8) with permission granted by Springer Nature].

Acquired Brown syndrome can result from orbital or strabismus surgery, sinusitis, systemic inflammatory disease, trauma, tumor, or manifest in association with trochleodynia (8, 23). It is believed that chronic trochleodynia has the potential to induce stenosing tenosynovitis of the SO muscle tendon and its sheath, subsequently leading to movement restriction in the inferior oblique (IO) field of action, with failure of the SO to relax/stretch and/or physical restriction of its tendon to slide through its tendon sheath (24). The latter may manifest as an audible click similar to the clinicopathogenesis of trigger finger. The hallmark of Brown syndrome is decreased or absent eye elevation in adduction without elevation deficits in abduction (23). The diagnosis of acquired Brown syndrome should be considered if this motility restriction exists in conjunction with clinical or imaging evidence of trochlear region inflammation. The representative motility deficits in Brown syndrome are depicted in Figure 3. Imaging features of a nodular SO tendon may be absent, and the background inflammation from trochleodynia is the most common imaging abnormality if present.

The presence of systemic inflammatory and connective tissue diseases increases the risk of BSAT, and this has been reported in association with enteropathic arthropathy (25), juvenile idiopathic arthritis (26), psoriasis (27), rheumatoid arthritis (28), Sjögren's syndrome (29), SLE (30–33), systemic sclerosis (34, 35), and AOSD (36). Patients may have either active SO inflammation (27, 29, 33, 34, 37) or unremarkable imaging presumably from resolved inflammation (28, 31, 35). Most of these case reports were written before trochleodynia was a recognized diagnosis; therefore, it is possible some of these patients presented with features of trochleodynia as well. Giannaccare et al. (8) found a small subset of patients they described as secondary to trochleitis, but there was no evidence to support that untreated trochleodynia led to acquired Brown syndrome, only that the features of both were present in their patients, hence our terminology BSAT.

### Differential Diagnosis

There are several entities that should be considered in a patient with trochleodynia (Table 2).

**Table 2:** Differential diagnoses.

	**Clinical presentation and history**	**Imaging findings**	**Lab findings**	**Typical treatment modalities**
Trochleodynia, primary idiopathic	1 Frontal headache from superonasal orbit spreading to ipsilateral periorbital borders and hemicranium 2 Point tenderness at trochlea, 3 Pain exacerbation due to heightened physical/emotional stress (chronic nerve trauma) or pain exacerbation with superior oblique muscle contraction or stretching (myofascial trigger point pathogenesis) Negatives: no autonomic signs such as conjunctival injection/lacrimation, nasal congestion/rhinorrhea, eyelid edema, forehead/fascial sweating, miosis/ptosis	No abnormal findings are possible; CT, MRI: thickening of trochlea and/or superior oblique tendon sheath with surrounding edema. A scan ultrasonography if technician is available	Idiopathic: no abnormalities	Trochlear injection of corticosteroid with local anesthetic leads to remission; trial of oral NSAIDs is acceptable with mild symptoms
Trochleodynia secondary to systemic inflammatory disease or trauma (6, 18, 19)	Trochleodynia may precede diagnosis of systemic disease which has been reported in granulomatosis with polyangiitis (GPA), systemic lupus erythematosus (SLE), incomplete Behçet's syndrome, orbital lymphoma, adult-onset Still's disease, Tolosa-Hunt syndrome	Same as in idiopathic trochleodynia	If present, laboratory abnormalities are consistent with the corresponding systemic disease, for example in SLE, patient may have (+)ANA, (+)anti-dsDNA, hypocomplementemia	Treatment of the systemic disease; may require trochlear injection of corticosteroid with local anesthetic
Brown syndrome (associated with trochleodynia) (8, 23–32, 34–36)	History of trochleodynia, trauma, strabismus surgery, sinusitis, or systemic inflammatory disease resulting in tenosynovitis of superior oblique tendon and restrictions as it moves through the sheath and trochlea 1 Decreased or absent passive or active elevation in adduction with normal elevation in abduction 2 Vertical diplopia in primary gaze (absent in trochleodynia) 3 Localized pain exacerbated by supraduction that is not associated with headache 4 Audible click may be present	CT or MRI: radiographic signs are not specific and may coincide with inflammation of trochleodynia	If present, laboratory abnormalities are consistent with the corresponding systemic disease	Treatment of systemic disease usually leads to improvement; trochlear injection of corticosteroid with local anesthetic; surgical intervention in refractory cases
Orbital myositis (29, 38, 39)	Often due to systemic inflammatory disease affecting superior oblique muscle but may be idiopathic: Periorbital pain with exacerbation by eye movement, may be associated with diplopia and proptosis, isolated superior oblique muscle is least frequently reported	CT or MRI: marked enlargement of the muscle(s) and possibly its tendon	WBC, ESR, CRP do not need to be elevated	Mainstay is 1 mg/kg/day oral prednisone or pulse IV methylprednisolone (39)
Thyroid ophthalmopathy with superior oblique involvement (40, 41)	Superior oblique overaction, incyclotorsion, vertical incomitance in horizontal gaze fields, other signs and symptoms of thyroid eye disease	CT or MRI: enlargement of superior oblique muscle usually along with inferior rectus or other recti muscles	Thyroid panel abnormalities, presence of anti-thyroid antibodies	Treatment of thyroid eye disease
Paroxysmal hemicrania and hemicrania continua (21, 22, 42)	Pain is strictly unilateral, orbital, supraorbital, or temporal; associated with autonomic signs ipsilateral to headache: conjunctival injection/lacrimation, nasal congestion/rhinorrhea, eyelid edema, forehead/fascial sweating, miosis/ptosis. Paroxysmal: 2–30 min severe attacks occurring >5 times per day or >20 attacks total. Continua: Less severe continuous (>3 months) pain with moderate or severe exacerbations	No abnormal findings at the trochlea	No abnormal findings	Oral NSAIDs (indomethacin) first line, trochlear corticosteroid injection may be needed for coexisting trochleodynia
Periorbital neuralgias (43–45)	Almost always associated with trauma or physical compression, manipulation: baseline pain with severe sharp pain during exacerbations, tenderness to palpation along path of supratrochlear, supraorbital, infraorbital nerves. Neuralgias can be overlapping or exist as isolated to one of these nerves. Negative: No pain exacerbation with vertical eye movements	No imaging features	No abnormal findings	Neuropathic pain oral drugs, Local anesthetic blockade
Cavernous sinus syndromes (46, 47)	Ocular, periorbital pain, proptosis from orbital congestion, ophthalmoplegia, miosis/mydriasis. Fistulas: ocular bruit, chemosis, conjunctival injection, diplopia Thrombosis: infectious process involving sinuses or orbital cellulitis, chemosis, conjunctival injection	MRI is diagnostic	CBC, ESR/CRP, and infectious workup identifies likely etiologies	Tumor: radiotherapy, stereotactic radiosurgery Fistulas: endovascular occlusion and carotid artery ligation Thrombosis: systemic antibiotics, corticosteroids, surgical drainage of abscess
Tolosa-Hunt syndrome (6, 48–50)	Unilateral periorbital or hemicranial pain with ipsilateral CN III, IV VI palsies, miosis, or ptosis, CN V1 sensory impairment	MRI: evidence of inflammation of cavernous sinus, superior orbital fissure, or orbit	No abnormal findings	Oral prednisolone

Orbital myositis often presents with painful ophthalmoplegia exacerbated with eye movements and orbital pain; the SO muscle is least likely to be involved (51) but has been reported (29, 38). The key difference is intense inflammation of the muscle belly usually detectable by imaging and relative sparing of the trochlea. The inflammation causes impairment or inability of the muscle to relax, limiting supraduction in adduction similar to acquired Brown syndrome (39).

In thyroid ophthalmopathy, there is no trochlear tenderness and the pain is a mild ache, which can be exacerbated by eye movements. The SO muscle can be enlarged and restricted (40, 41), but the recti are virtually always enlarged and restricted as well. This along with other ophthalmic findings commonly present in thyroid ophthalmopathy such as proptosis and lid retraction make distinguishing this diagnosis from trochleodynia straightforward. The SO enlargement leads to overaction of the SO with a similar pattern of ophthalmoplegia to Brown syndrome including impaired elevation in adduction but with associated prominent incyclotorsion (40). Rarely trochleodynia has been reported following orbital decompression surgery for Graves' ophthalmopathy (6).

Autonomic signs such as lacrimation, conjunctival injection, rhinorrhea, nasal congestion, and miosis/mydriasis are not associated with trochleodynia. If present, trigeminal autonomic cephalalgias (TACs) should be considered and include cluster headaches, paroxysmal hemicrania and hemicrania continua among others. Cluster headaches cause peri-orbital pain during attacks but are easily differentiated from trochleodynia given the presence of autonomic derangements ipsilateral to the pain (52). Paroxysmal hemicrania and hemicrania continua share these similar autonomic signs. According to the International Classification of Head Disorders 3rd Edition, paroxysmal hemicrania's features include severe, unilateral paroxysms of pain in orbital, supraorbital, temporal areas lasting 2–30 min multiple times per day with pain free episodes. Hemicrania continua is differentiated by temporality in which pain is less severe and continuous with intermittent episodes similar to paroxysmal hemicrania. Trochleodynia can coexist with these particular TACs. This is significant because control of trochleodynia is critical to remission of both coexisting headaches. A 60 year-old woman developed trochleodynia after her paroxysmal hemicrania was under control with indomethacin (21). Triamcinolone and lidocaine local injection led to remission of her trochleodynia. A 53 year-old woman was diagnosed with hemicrania continua but could not be managed with indomethacin due to allergic reaction; control of coexisting trochleodynia with triamcinolone injection led to remission of both (22).

Periorbital neuralgias of clinical significance include supraorbital, supratrochlear and infratrochlear. These are often associated with previous trauma, long-term compression such as helmet use, or cranial surgeries proximal to the course of these nerves. However, primary idiopathic cases exist. Baseline pain generally follows nerve topography involving the forehead, eyebrow and internal angle of the orbit that is constant with exacerbations experienced transiently as severe pain with a sharp, shock-like quality. Pain can be elicited with pressure at the supraorbital notch (supraorbital) (43), medial third of supraorbital rim (supratrochlear) (44), or internal angle of the orbit above medial canthus (infratrochlear) (45). A key difference from trochleodynia is lack of pain exacerbation with vertical eye movements or ophthalmoplegia. Periorbital neuralgias respond well to oral medications used for neuropathic pain, such as gabapentin, and local anesthetic blockade (43–45). Lacrimal and infraorbital nerve neuralgias have clearly distinct pain topography from trochleodynia (53, 54) and are not further discussed.

Although unlikely to be mistaken for trochleodynia, cavernous sinus syndromes from inflammatory, malignant, and infectious etiologies may present with peri-orbital pain with ophthalmoplegia, anisocoria, proptosis due to orbital congestion, and trigeminal sensory loss (46, 47). Isolated CN IV involvement is highly unlikely, there is no trochlear tenderness, and the constellation of findings would be explained with MRI of brain and orbits. Treatment depends on etiology. Inflammation of the cavernous sinus often referred to by the eponym, Tolosa-Hunt syndrome, is highly responsive to systemic corticosteroids (48, 49). Tolosa-Hunt is usually distinguishable clinically from trochleodynia and we would not recommend neuroimaging for trochlear region pain in the absence of a cranial nerve palsy.

**Figure 4:**
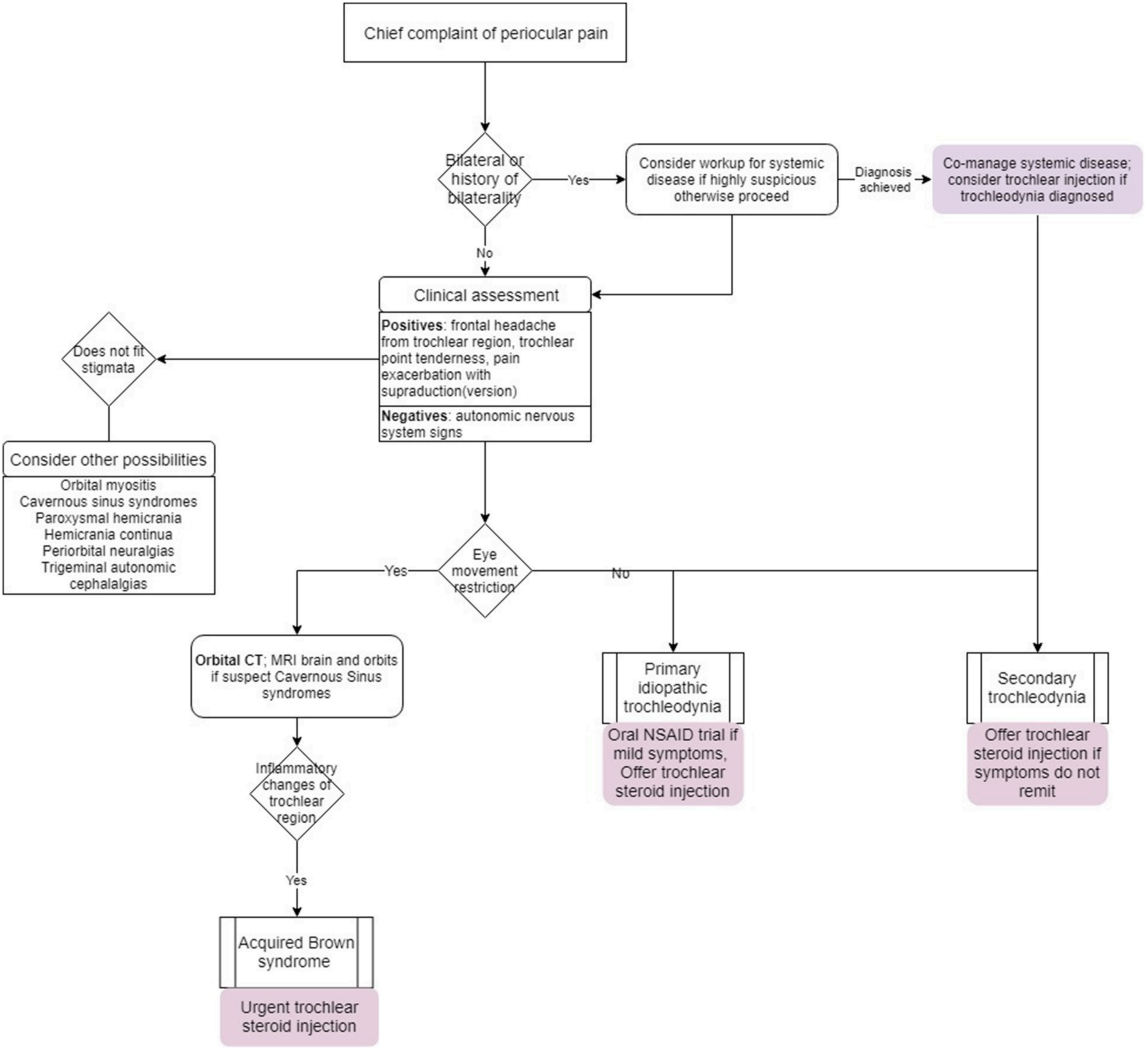
Algorithm for trochleodynia workup and management.

A proposed algorithm for evaluation and management is depicted in Figure 4.

## Management

**Figure 5:**
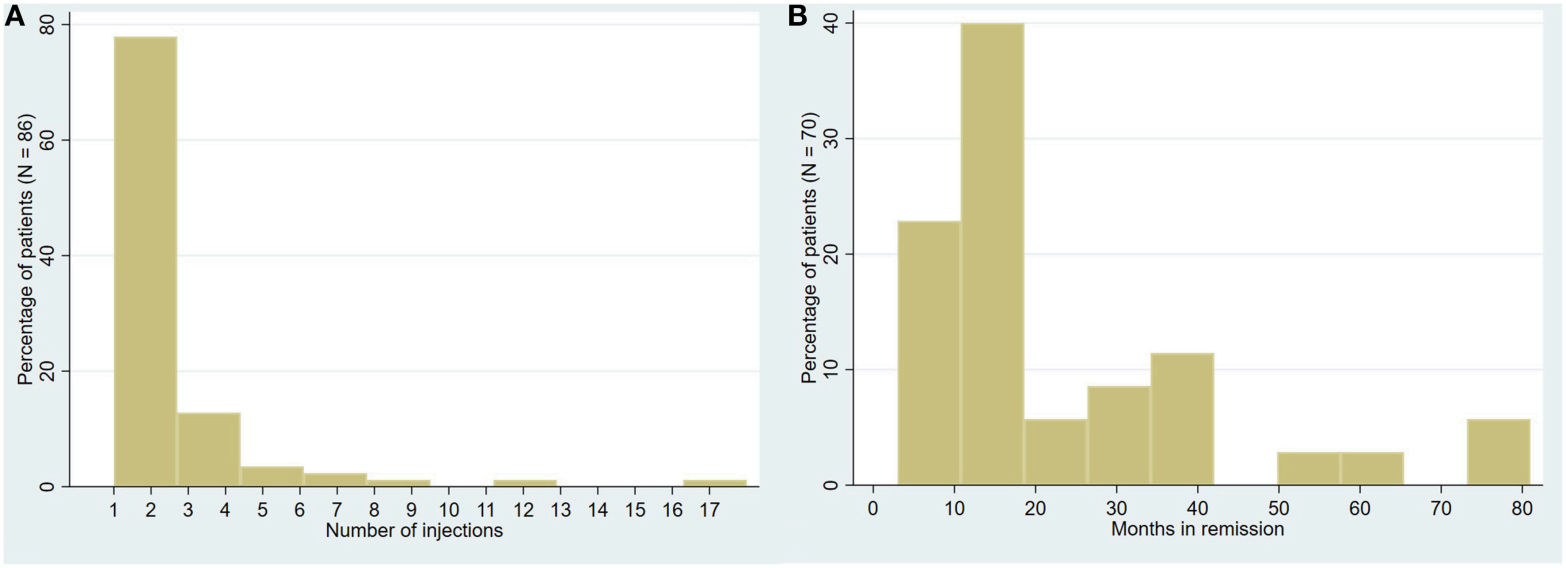
Trochleodynia patients receiving injections. **(A)** Distribution of total number of injections administered (pooled among 86 patients). **(B)** Distribution of remission period post-effective injection therapy (pooled follow-up data among 70 patients with reported follow-up).

Reviewing the English language literature, we have combined all identified case series (5, 6, 8–12) and case reports (18–22) of patients with the diagnosis of trochleodynia to perform a meta-analysis (Table 3). Descriptive statistics and significance tests were performed in STATA 14 (StataCorp, College Station, TX, USA). Consisting of 181 patients with a mean age of 43.7 (SD 18.3) years, the vast majority, 83.4%, were female. There has only been one reported pediatric case (55). Acquired Brown syndrome was also diagnosed in 10.5% of cases. Less than half (47.5%) of patients were managed with local corticosteroid injections with an average count of 2.2 injections each (Figure 5A). Injection patients achieved an average remission period of 18 months [range 0–18 months] (Figure 5B). The remission period was determined by aggregating total follow-up time reported by the authors; in cases of multiple injections, the remission period was assumed as total time following the most recent injection unless otherwise informed. There were cases where a patient had no recurrence with a reported follow-up time. This value was used despite the remission period potentially being much longer as there was no more follow-up data. Given these inherent limitations of aggregating data from retrospective cohorts from varied time periods, it was not possible to draw further conclusions such as what diagnoses, co-morbidities, or predisposing demographic factors were associated with better treatment outcomes let alone compare oral vs. local corticosteroid injection. In general, patients failed oral therapy with NSAIDs, anti-depressants, anti-convulsants, opioids, and steroids; the patients were then offered injections. However, the two more recent cohorts from Jarrin et al. and Chanlalit et al. reported a trial of oral NSAIDs and use of injections only if symptoms were not controlled (9, 10).

**Table 3:** Meta-analysis of retrospective case series and case reports.

**Report (*N* = sample size)**	**Age (mean ± SD)**	**%Female**	**Bilateral *N* (%)**	**Acquired Brown syndrome**	**Comorbid headaches**	**Injection patients *N* (% of all cases)**	**Average injections**	**Average remission period for injections (months)**
Tychsen et al. (*N* = 13) (11)	47.6 ± 16.2	69	0 (0%)		NR	2 (15%)	1	6
Yanguela et al. (*N* = 5) (12)	53.2 ± 12.1	100	0 (0%)		MH 5 (100%)	4 (80%)	NR	11.2
Yanguela et al. (*N* = 18) (5)	44.8 ± 13.5	94	3 (16.6%)		MH 10 (55.6%) TTH 1 (5.6%)	16 (88%)	NR	8.35
Zaragoza-Casares et al. (20)	23	100	1 (100%)		MH 1 (100%)	1 (100%)	1	NR
Pego-Reigosa et al. (21)	60	100	0 (0%)		0 (0%)	1 (100%)	2	4
Cuadrado et al. (22)	53	100	0 (0%)		0 (0%)	1 (100%)	1	3
Fonseca et al. (18)	26	100	1 (100%)		MH 1 (100%)	1 (100%)	2+ (total NR)	NR
Gutmark et al. (19)	23	0	1 (100%)		0 (0%)	0 (0%)	0	NA
Smith et al. (*N* = 25) (6)	47.3 ± 15.7	80	11 (44%)		MH 7 (28.0%) TTH 1 (4.0%)	25 (100%)	4	22.5
Giannaccare et al. (*N* = 13) (8)	30.4 ± 26.6	54	0 (0%)	13 (100%)	0 (0%)	13 (100%)	1.5	32.9
Jarrin et al. (*N* = 59) (9)	43 ± 18	86.4	1 (1.7%)	6 (10.2%)	0 (0%)	8 (13.5%)	1.4	NR
Chanlalit et al. (*N* = 43) (10)	Median: 51 Range: 18–88	88.3	21 (48.8%)		MH 2 (4.6%) TTH 3 (7.0%)	14 (36%)	1	11
	43.7 ± 18.3	83.4	19.9% (95%CI 14.7–26.4)	10.5%	MH 15.5% (95%CI 10.7–21.8) TTH 3.0% (95%CI 1.2–7.0)	86 (47.5%)	2.2 [range 1–18]	18.0 [range 0–81]

*MH, migraine headache; NA, not applicable; NR, not reported; TTH, tension-type headache*.

In patients who are treatment naïve with mild, recent-onset symptoms, no coexisting headache disorders, no imaging evidence of inflammation, no diplopia, or ophthalmoplegia, it is probably reasonable to trial oral NSAIDs. Follow-up is by clinical judgement during initial assessment and it may take up to 21 days for response (10). If the patient has MH or TTH, offering local corticosteroid injection would be reasonable given trochleodynia's likely contribution to poor control of coexisting headache disorders. Yanguela et al. achieved response to injection in 95% within 48 h; 70% of patients with coexisting headache disorders reported improvements with decreased basal pain, exacerbation frequency, and need for analgesics (5).

When pain is more severe and acute, particularly if associated with Brown syndrome, local corticosteroid injection is indicated (8). Giannaccare et al. achieved significant improvement of pain symptoms within 5.53 ± 1.78 days and within 22.45 ± 13.85 days, their patients were in complete remission including absence of diplopia in primary gaze. At average follow-up duration of 32.9 months, all their injection patients were in full remission. If a patient has a diagnosis of BSAT, it is important to offer local corticosteroid injection as early as possible (7.8 days average from diagnosis to injection in Giannaccare et al. (8). It is believed some individuals experience a robust inflammatory response necessitating multiple rounds of injections or ultimately end in treatment failure. In these cases, the permanent ophthalmoplegia and resulting diplopia require surgical correction. Operative techniques for acquired Brown syndrome are beyond the scope of this review.

Unfortunately, most patients sit in a gray area between the two aforementioned clinical stages and may not require injection immediately if symptoms are moderate. We propose that if imaging shows evidence of inflammation affecting the SO tendon/tendon sheath in addition to the trochlea, local corticosteroid injection should be strongly considered. If the patient has bilateral symptoms, an underlying systemic inflammatory condition could be considered, especially if history elicits systemic symptoms and signs. Treatment of the underlying systemic disease should also treat the trochleodynia. If there are signs or symptoms of persistent trochleodynia following systemic disease treatment, local corticosteroid injection should be offered. There is no role for oral corticosteroids unless it is needed for an underlying systemic disease. In all the case series and case reports, oral corticosteroids were not associated with remission of primary idiopathic trochleodynia. In regard to neuropathic and neuromuscular etiologies of pain, gabapentin has been used with limited to negligible efficacy (6).

### Corticosteroid Injection

**Figure 6:**
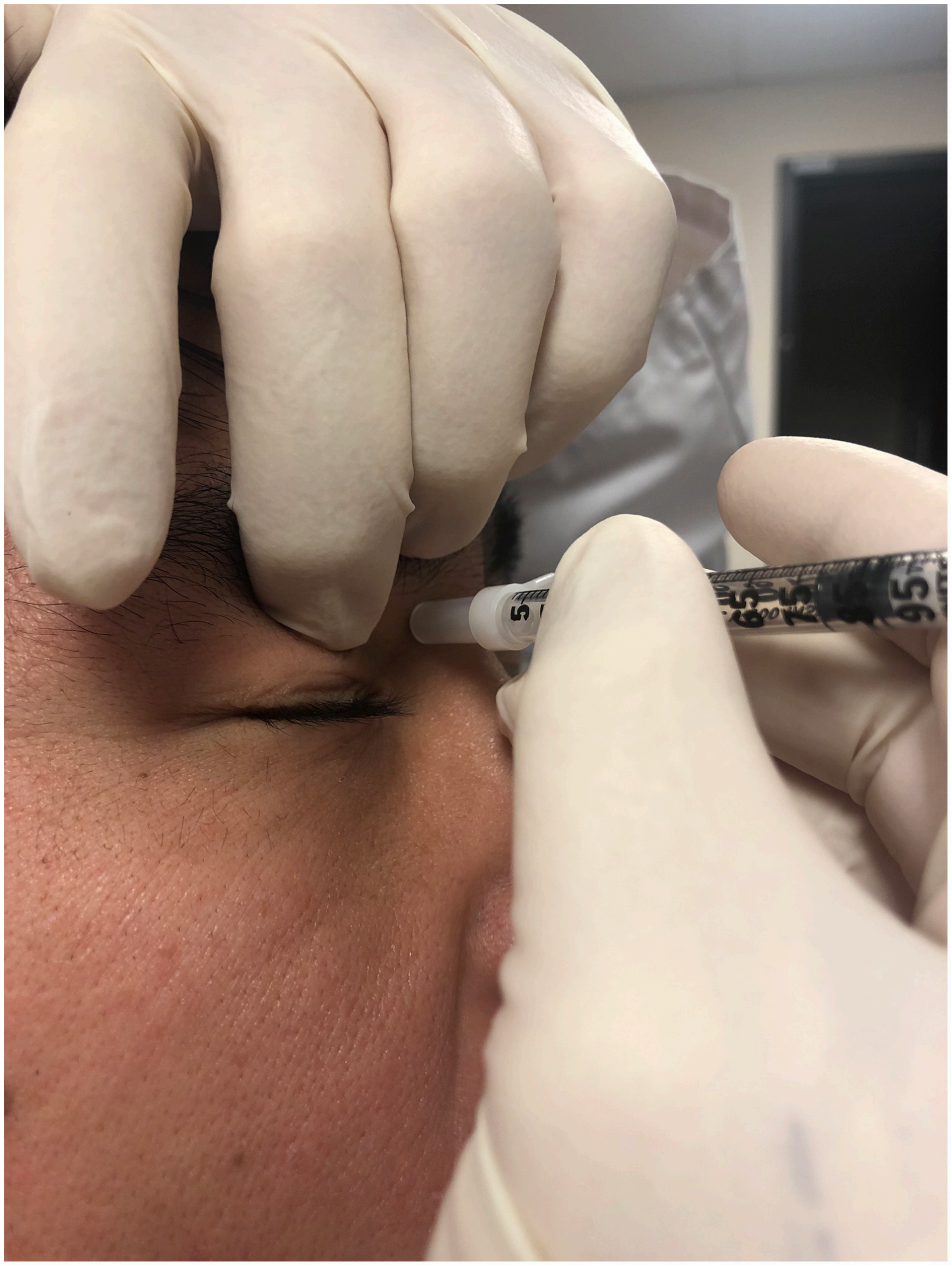
Site for trochlear injection. Ideally, the index finger of the non-injecting hand pushes the globe down and out to make more room for the injection. The aimed site is right below the trochlea and not the trochlea itself. The needle is angled away from the globe.

There is no standard dose, but most authors would agree 1–3 mg dexamethasone with lidocaine can be offered at each injection; alternatively, up to 40 mg triamcinolone can be used. The ideal injection site is depicted in Figure 6. A short, thinner (30-gauge) needle is ideal for corticosteroid solutions, but suspensions require a larger bore (25-gauge) because the particulate can clog the needle. Performed properly, the risk of globe perforation should be virtually zero since aiming for the trochlea directs the needle away from the globe. Among the 187 injections reported, complications included two cases of local bruising, one injection site hematoma, three peri-trochlear hemorrhages, and one likely incidental otitis for a total complication rate of 3.2% (95%CI 0.5–5.9) (5, 6, 10, 12). All hematologic complications were self-resolving; the otitis was successfully treated with local antibiotics (5). Temporary diplopia from SO anesthesia is possible, and the authors know of one patient who was diagnosed with an acquired Brown syndrome as a complication of intra-trochlear injection (personal correspondence by the authors).

Use of periocular steroid injections in treatment of thyroid eye disease show similar safety profile, with notable absence of vascular occlusion, intraocular pressure elevation, corneoscleral melting, or fat atrophy (56–58). Nevertheless, there has been one recent case report of a central retinal artery occlusion after a 20 mg injection of triamcinolone for thyroid ophthalmopathy (59). If injected into a vessel anastomotic with the ophthalmic artery, the large particle size of triamcinolone (1–1,000 μm) poses a risk of occluding retinal arterioles. Dexamethasone is a non-particulate steroid and is safer in this regard, but theoretically does not control symptoms as long as suspensions. No consistent meaningful difference in trochleodynia symptom remission has been reported between particulate and non-particulate corticosteroid injections. Symptoms should improve over 3–7 days following the injection. Our general approach is to repeat injections more than 30 days later. The wide variability in treatment response is unclear. In Smith et al.'s series, a 57 year-old female needed one injection for complete remission, while a 42 year-old female responded by the 17th and 18th injection with 5–7 months lasting effect (6); both patients were diagnosed with primary trochleodynia without co-existing headaches.

## Conclusion

Trochlear pain (trochleodynia) is becoming recognized as a set of disorders that can present in isolation or concomitantly with co-existing migraines, tension-type headaches, or other headache disorders, possibly explaining subpar symptom control in a small but significant number of individuals globally. Trochleodynia features unilateral periocular pain that may involve the ipsilateral hemicranium. Pain exacerbation occurs with trochlear palpation and supraduction of the affected eye especially in the adducted position. Trochleodynia may respond to oral NSAIDs if symptoms are mild and of recent onset. While oral NSAIDs may lead to remission with moderate to severe symptoms, the patient should be offered trochlear injection of corticosteroids. Bilateral manifestations could be concerning for an underlying systemic inflammatory disease, and workup could be considered (Table 1). Control of associated underlying disease almost always leads to trochleodynia remission. Nevertheless, in order to identify the optimal treatment paradigm for trochleodynia and better understand variations in response to treatment, prospective randomized control trials are required.

## Author Contributions

All authors listed have made a substantial, direct and intellectual contribution to the work, and approved it for publication.

### Conflict of Interest Statement

The authors declare that the research was conducted in the absence of any commercial or financial relationships that could be construed as a potential conflict of interest.
